# vNOTES Versus Laparoscopic Lateral Suspension for Advanced Pelvic Organ Prolapse: A Single-Center, Single-Surgeon Retrospective Cohort Study

**DOI:** 10.3390/jcm15187250

**Published:** 2026-09-18

**Authors:** Serhat Sarıkaya, Yunus Emre Purut, Anıl Doğukan Tutal, Ceren Sağlam Purut, Göktürk Han Doğan, Sercan Kantarcı, Adnan Budak

**Affiliations:** 1 Department of Obstetrics and Gynecology, University of Health Sciences, İzmir Tepecik Training and Research Hospital, 35180 Izmir, Türkiye; 2 Department of Perinatology, İzmir Atatürk Training and Research Hospital, 35150 Izmir, Türkiye; drcerensaglam@yahoo.com

**Keywords:** laparoscopy, pelvic organ prolapse, postoperative pain, quality of life, surgical mesh, vaginal natural orifice transluminal endoscopic surgery

## Abstract

**Background/Objectives**: Vaginal natural orifice transluminal endoscopic surgery lateral suspension (vNOTES-LS) may reduce the perioperative burden of conventional laparoscopic lateral suspension (LLS), but comparative evidence remains limited. We compared perioperative, anatomical, safety, and patient-reported outcomes. **Methods**: This single-center retrospective cohort included 97 women with stage III–IV pelvic organ prolapse who underwent vNOTES-LS (*n* = 33) or LLS (*n* = 64) and completed 12-month follow-up. The primary outcome was anatomical success at 12 months. **Results**: Operating time was shorter with vNOTES-LS (78.0 ± 16.6 vs. 110.0 ± 14.2 min; *p* < 0.001), as were estimated blood loss, hospitalization, and 24-h pain scores. After adjustment, vNOTES-LS was associated with a 31.7-min shorter operating time (95% CI, −38.7 to −24.6; *p* < 0.001). Anatomical success was 87.9% versus 89.1% (*p* = 1.000; RR, 0.99; 95% CI, 0.85–1.15), with an absolute risk difference of −1.2 percentage points (95% CI, −14.7 to 12.3). Cumulative mesh exposure within 12 months was 9.1% and 9.4%, respectively. No statistically significant between-group differences were detected in recurrence, measured safety outcomes, or longitudinal changes in the Pelvic Floor Distress Inventory-20 (PFDI-20), Pelvic Floor Impact Questionnaire-7 (PFIQ-7), and 12-item Pelvic Organ Prolapse/Urinary Incontinence Sexual Questionnaire (PISQ-12). **Conclusions**: vNOTES-LS was associated with shorter operating time and favorable early perioperative outcomes. Observed 12-month anatomical success rates were not statistically significantly different; however, equivalence or non-inferiority cannot be concluded.

## 1. Introduction

Pelvic organ prolapse (POP) is characterized by the descent of one or more pelvic organs into or through the vaginal canal as a consequence of impaired connective-tissue and muscular support. Advanced POP may cause vaginal bulging, pelvic pressure, urinary and bowel symptoms, sexual dysfunction, and substantial impairment of quality of life. The Pelvic Organ Prolapse Quantification (POP-Q) system provides a standardized and reproducible method for describing the anatomical severity and compartmental distribution of prolapse [1]. Although conservative treatment may be appropriate for selected patients, surgical reconstruction is frequently required for symptomatic stage III or IV disease.

Adequate apical support is a central component of reconstructive prolapse surgery because failure to restore apical support may contribute to persistent or recurrent anterior and posterior compartment prolapse. Laparoscopic lateral suspension (LLS), initially described as a mesh-based alternative for anterior and apical prolapse, provides bilateral tension-free support while avoiding dissection and fixation at the sacral promontory [2]. Subsequent studies using standardized techniques have reported favorable anatomical outcomes and acceptable morbidity, supporting LLS as an established minimally invasive option for advanced anterior and apical prolapse [3]. Nevertheless, conventional LLS requires abdominal trocar placement and laparoscopic access, which may contribute to postoperative pain, visible abdominal scars, and a longer recovery period.

Vaginal natural orifice transluminal endoscopic surgery (vNOTES) enables endoscopic access to the pelvic cavity through a transvaginal route and may reduce the abdominal access–related burden of conventional laparoscopy. The adaptation of lateral suspension to the vNOTES approach was described with the Salman–Ketenci Gencer technique, which applies the anatomical principles of lateral mesh suspension without conventional abdominal laparoscopic trocar placement [4]. Recent comparative studies have reported favorable short-term outcomes with vNOTES-LS. A retrospective cohort found a shorter hospital stay but no significant difference in operative time, whereas a prospective cohort reported both shorter operative time and shorter hospital stay with vNOTES-LS [5,6].

However, the available comparative evidence remains limited. Published studies have generally included relatively small cohorts, heterogeneous surgical protocols, or short follow-up periods, and few have simultaneously assessed compartment-specific anatomical recurrence, perioperative recovery, procedure-related complications, sexual function, and validated patient-reported outcomes. Furthermore, evidence derived from a single-center and single-surgeon setting may help reduce the influence of institutional and technical variability when comparing the two approaches.

The present study therefore aimed to compare vNOTES-LS and conventional LLS for the treatment of advanced pelvic organ prolapse in a single-center, single-surgeon retrospective cohort. The primary outcome was anatomical success at 12 months. Secondary outcomes included perioperative measures, compartment-specific recurrence, safety outcomes, pelvic floor symptom burden, quality of life, sexual function, and patient-perceived global improvement. The study was designed to compare perioperative, anatomical, safety, and patient-reported outcomes between the two surgical approaches without a prespecified non-inferiority or equivalence hypothesis.

## 2. Materials and Methods

### 2.1. Study Design and Setting

This retrospective comparative cohort study included consecutive women who underwent surgical treatment for advanced pelvic organ prolapse at the Department of Obstetrics and Gynecology, University of Health Sciences, İzmir Tepecik Training and Research Hospital, İzmir, Türkiye, between January 2024 and June 2025. The study was conducted at a single tertiary teaching and research hospital, and all surgical procedures were performed by the same surgeon (S.S.), who had 11 years of specialist experience in obstetrics and gynecology and approximately 8 years of experience performing conventional laparoscopic lateral suspension. vNOTES-LS was introduced into his clinical practice in August 2024. During the study period, 39 vNOTES-LS procedures were performed, and by the time of manuscript revision, the surgeon’s cumulative experience with vNOTES-LS had reached approximately 70 procedures. Following the introduction of vNOTES-LS, both vNOTES-LS and conventional LLS continued to be performed contemporaneously throughout the remainder of the study period. Postoperative follow-up data through June 2026 were reviewed.

Eligible patients were classified according to the surgical approach as vaginal natural orifice transluminal endoscopic surgery lateral suspension (vNOTES-LS) or conventional laparoscopic lateral suspension (LLS). The choice of surgical route was individualized according to pelvic anatomy, previous surgical history, patient preference, and surgeon judgment. No random allocation was performed.

Ethical approval for this retrospective study was obtained from the İzmir Provincial Health Directorate, University of Health Sciences İzmir Tepecik Training and Research Hospital Non-Interventional Research Ethics Committee (decision no. 2026/04-51; 7 May 2026). The study was conducted in accordance with the Declaration of Helsinki. Written informed consent had been obtained from all patients before surgery.

### 2.2. Study Population

**Figure 1:**
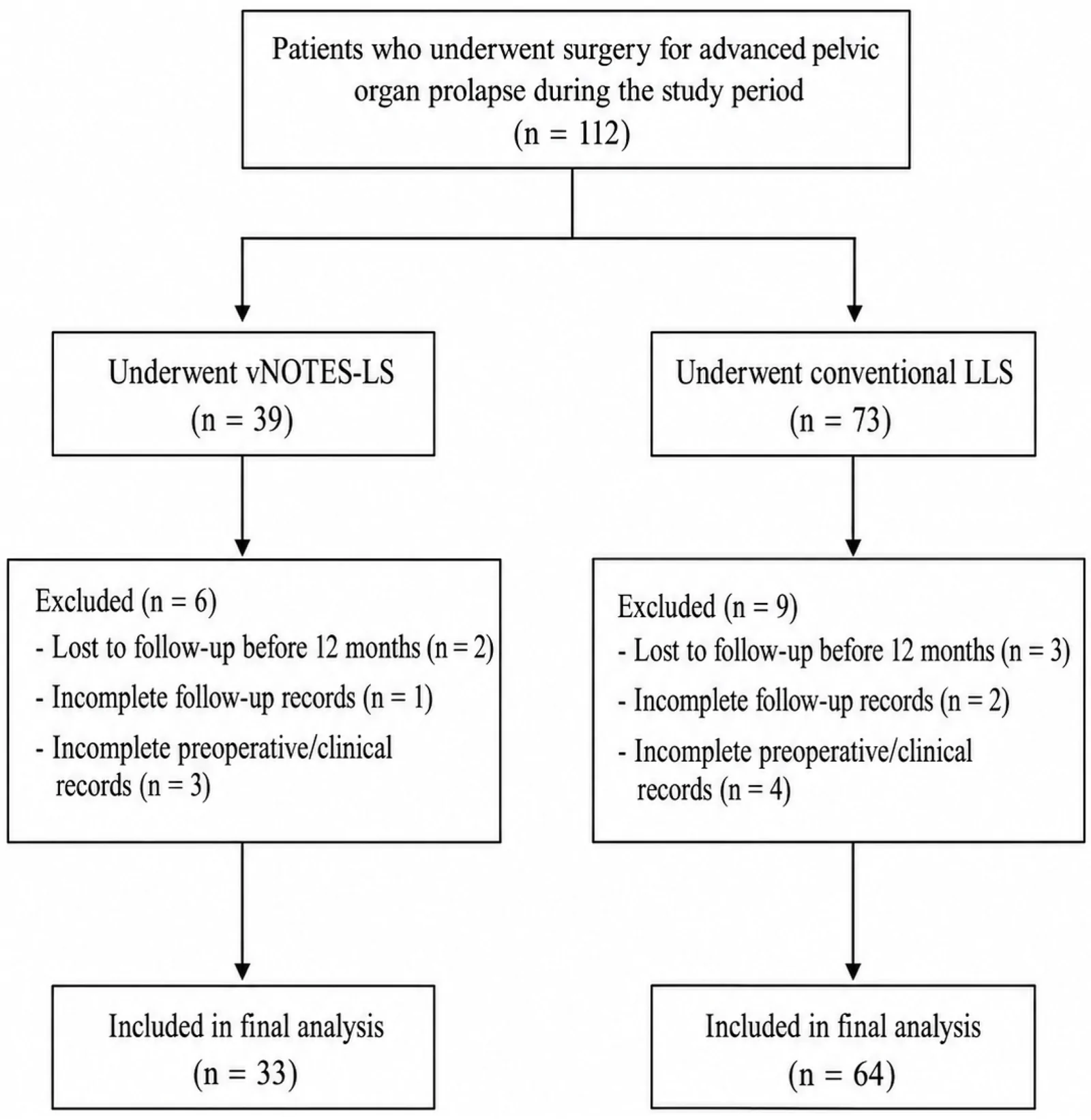
Flow diagram of patient selection and follow-up. LLS, laparoscopic lateral suspension; vNOTES-LS, vaginal natural orifice transluminal endoscopic surgery lateral suspension.

Women were eligible for inclusion if they had symptomatic stage III or IV pelvic organ prolapse, underwent vNOTES-LS or conventional LLS during the study period, and had sufficient preoperative, perioperative, and follow-up data for evaluation of the prespecified outcomes. Patients were excluded if they were lost to follow-up before completion of the 12-month assessment or had incomplete preoperative, clinical, or follow-up records that precluded evaluation of the study outcomes. The patient-selection process is summarized in Figure 1.

Demographic and clinical variables included age, body mass index, parity, number of vaginal births, menopausal status, sexual activity, smoking status, diabetes mellitus, hypertension, constipation, previous prolapse surgery, previous stress urinary incontinence surgery, dominant prolapse compartment, and concomitant pelvic floor procedures.

Pelvic organ prolapse was evaluated using the standardized Pelvic Organ Prolapse Quantification (POP-Q) system. POP-Q measurements were recorded in centimeters relative to the hymen, and prolapse severity was classified according to the standardized staging system described by Bump et al. [1].

### 2.3. Surgical Procedures

Both procedures were performed under general anesthesia using a standardized institutional surgical pathway. A 30 × 30-cm polypropylene mesh (Paha^®^ Polypropylene Mesh, P3030; Atiyalar Medikal, Ankara, Türkiye) was manually tailored intraoperatively into a V-shaped configuration with approximately 2-cm-wide lateral arms. Mesh fixation was performed using approximately 6–7 interrupted sutures with a combination of 2-0 polyglactin 910 (Vicryl^®^; Ethicon, Raritan, NJ, USA) and 2-0 polypropylene (Prolene^®^; Ethicon, Raritan, NJ, USA).

Conventional laparoscopic lateral suspension was performed according to the principles of the Dubuisson technique [2,3]. After conventional laparoscopic abdominal access was established, the vesicovaginal space and apical support area were prepared according to the patient’s anatomy and the planned concomitant procedures. For lateralization of the mesh arms, small bilateral skin incisions were made approximately 2 cm above the iliac crest and 4–5 cm posterior to the anterior superior iliac spine. A grasping forceps was advanced through a retroperitoneal/subperitoneal tunnel toward the region of the round ligament under laparoscopic visualization, and each lateral mesh arm was drawn through this tunnel toward the corresponding abdominal wall incision. After appropriate tension-free positioning, the mesh was completely peritonealized.

In the vNOTES-LS group, the lateral suspension procedure was performed according to the technique described by Salman and Ketenci Gencer [4]. No dedicated vNOTES access platform was used. Following completion of vaginal hysterectomy with LigaSure™ Small Jaw Open Sealer/Divider (Medtronic, Minneapolis, MN, USA) for pedicle control, the central portion of the V-shaped polypropylene mesh was positioned in the vesicovaginal plane between the bladder and vaginal cuff, approximately 2 cm below the cuff line, and fixed to the anterior apical vaginal support area using approximately 6–7 interrupted sutures with a combination of 2-0 polyglactin 910 (Vicryl^®^; Ethicon, Raritan, NJ, USA) and 2-0 polypropylene (Prolene^®^; Ethicon, Raritan, NJ, USA). The vaginal cuff was kept open during the lateral suspension maneuver. A 10-mm laparoscopic port (Applied Medical, Rancho Santa Margarita, CA, USA) was then introduced through the open cuff, and a laparoscopic camera inserted through this port provided direct endoscopic visualization of the pelvic cavity.

For lateralization of the mesh arms, small bilateral skin incisions were made approximately 2 cm above the iliac crest and 4–5 cm posterior to the anterior superior iliac spine. Under direct endoscopic visualization through the transvaginal 10-mm port, a grasping forceps was introduced from each lateral abdominal incision and advanced medially toward the pelvis through a subperitoneal/retroperitoneal tunnel. The tip of the grasper was advanced toward and delivered through the open vaginal cuff. The corresponding lateral arm of the previously fixed mesh was then loaded onto the grasper. The grasper was subsequently withdrawn laterally along the same subperitoneal/retroperitoneal tunnel toward the abdominal wall, thereby drawing the mesh arm from the pelvis to the external suspension point. The same maneuver was repeated on the contralateral side.

The lateral mesh arms were progressively drawn outward and tensioned under endoscopic visualization until satisfactory, symmetric, and tension-free apical support was achieved. The excess externalized mesh was then trimmed. Thus, the final length of each lateral arm was determined individually according to patient anatomy and the degree of suspension required rather than being predetermined. The central portion of the mesh remained within the vesicovaginal extraperitoneal plane, while both lateral arms were routed through subperitoneal/retroperitoneal tunnels. Thus, after completion of the procedure and peritoneal closure, the entire mesh was excluded from the intraperitoneal cavity. Following completion of bilateral suspension and confirmation of the desired apical support, the vaginal cuff was closed with a continuous 0 or 1 polyglactin 910 suture (Vicryl^®^; Ethicon, Raritan, NJ, USA) incorporating the peritoneum into the closure.

In uterus-preserving procedures, transvaginal access was obtained through an anterior colpotomy, and the central portion of the mesh was fixed to the anterior surface of the uterus using approximately 6–7 interrupted sutures with the same suture materials described above; the same bilateral subperitoneal tunneling and mesh-arm retrieval principles were then applied. When clinically indicated, additional pelvic floor procedures, including anterior colporrhaphy, posterior colporrhaphy, transobturator tape placement, and perineoplasty, were performed concomitantly.

### 2.4. Outcome Measures

The primary outcome was anatomical success at 12 months. Anatomical success was defined as the absence of prolapse reaching or extending beyond the hymen in any vaginal compartment and the absence of reoperation for recurrent prolapse. Patients who underwent reoperation for recurrent prolapse during follow-up were classified as anatomical failures regardless of subsequent postoperative POP-Q findings. POP-Q measurements were obtained during standardized postoperative pelvic examination and recorded in centimeters relative to the hymen. All preoperative and postoperative POP-Q examinations were performed by the same surgeon/examiner (S.S.) using the same standardized assessment technique. Accordingly, all compartment-defining POP-Q points were required to remain proximal to the hymen (<0 cm) for the patient to meet the anatomical success criterion. For secondary descriptive analysis, the same anatomical success criterion was also applied at the 6-month assessment. Additional secondary anatomical outcomes included postoperative POP-Q stage and apical, anterior, and posterior compartment recurrence at 6 and 12 months. Compartment-specific recurrence was determined according to the clinically documented recurrent compartment using the corresponding POP-Q measurements together with findings from the postoperative pelvic examination.

Perioperative outcomes included operating time, estimated blood loss, postoperative hemoglobin decrease, length of hospital stay, and pain intensity 24 h after surgery. Postoperative pain was assessed using a 10-point visual analog scale, with 0 indicating no pain and 10 indicating the worst imaginable pain.

Safety outcomes were evaluated at both 6 and 12 months and included mesh exposure, pelvic pain, dyspareunia, de novo stress urinary incontinence, urgency, voiding dysfunction, and reoperation for recurrent prolapse. Mesh exposure–related interventions were recorded separately as part of the management of mesh exposure. Mesh exposure was identified during postoperative pelvic examination and recorded according to its timing, size, location, associated symptoms, management, and subsequent clinical course. Cumulative mesh exposure within 12 months was defined as exposure diagnosed at any time during the first postoperative year, whereas active mesh exposure at the 12-month examination was defined as exposure that remained present or was newly identified at the 12-month assessment.

### 2.5. Patient-Reported Outcomes Measures

Pelvic floor symptoms and their effects on quality of life were evaluated preoperatively and at 6 and 12 months using the Pelvic Floor Distress Inventory-20 (PFDI-20) and Pelvic Floor Impact Questionnaire-7 (PFIQ-7) [7]. Validated Turkish versions of both questionnaires were used [8]. Lower PFDI-20 and PFIQ-7 scores indicate a lower symptom burden and less impairment of quality of life.

Sexual function was evaluated using the Pelvic Organ Prolapse/Urinary Incontinence Sexual Questionnaire-12 (PISQ-12) [9], using its validated Turkish version [10]. Because the PISQ-12 was designed for sexually active women, this outcome was analyzed only among participants who were sexually active at baseline and had complete measurements at all three assessments. Higher PISQ-12 scores indicate better sexual function.

Patient-perceived global improvement was evaluated at 6 and 12 months using the 7-point Patient Global Impression of Improvement (PGI-I) scale [11]. PGI-I scores of 1 (‘very much better’) or 2 (‘much better’) were classified as PGI-I-defined subjective success.

### 2.6. Statistical Analysis

The distribution of continuous variables was assessed using visual methods and the Shapiro–Wilk test. Continuous variables are presented as mean ± standard deviation or median with interquartile range, according to their distribution. Categorical variables are presented as number and percentage.

Between-group comparisons were performed using Welch’s independent-samples *t* test for approximately normally distributed continuous variables and the Mann–Whitney U test for non-normally distributed continuous variables. Categorical variables were compared using Pearson’s chi-square test or Fisher’s exact test, as appropriate.

Changes in PFDI-20, PFIQ-7, and PISQ-12 scores from baseline to 6 and 12 months were evaluated using two-way mixed repeated-measures analysis of variance. Time was included as the within-subject factor, and surgical approach was included as the between-subject factor. The time-by-group interaction was used to determine whether the magnitude of change differed between the two surgical approaches. PISQ-12 analyses were restricted to women who were sexually active at baseline and had complete measurements at all three time points. Between-group comparisons of baseline-to-12-month change scores were performed using Welch’s independent-samples *t* test.

A multivariable linear regression model was constructed to evaluate the association between surgical approach and operating time after adjustment for potential confounders. Surgical approach was entered as the primary explanatory variable. The model included age, body mass index, parity, preoperative POP-Q stage, concomitant hysterectomy, and the presence of any additional pelvic floor procedure. These covariates were selected on the basis of clinical relevance and their plausible association with operative complexity and duration rather than solely according to univariable statistical significance. Heteroscedasticity-consistent standard errors were used to account for potential non-constant error variance. Adjusted regression coefficients are reported with 95% confidence intervals.

Because only 11 anatomical failures occurred at 12 months, a fully adjusted multivariable model for the primary anatomical outcome was considered vulnerable to overfitting and unstable estimation. Accordingly, between-group anatomical success was described using unadjusted relative and absolute effect estimates with 95% confidence intervals rather than an overparameterized multivariable model.

The relative risk and absolute risk difference for 12-month anatomical success were calculated with 95% confidence intervals. All statistical analyses were performed using IBM SPSS Statistics for Windows, version 25.0 (IBM Corp., Armonk, NY, USA). All statistical tests were two-sided, and a *p* value < 0.05 was considered statistically significant.

## 3. Results

### 3.1. Study Population and Baseline Characteristics

During the study period, 39 women underwent vNOTES-LS and 73 underwent conventional LLS. In the vNOTES-LS group, 2 patients were lost to follow-up before the 12-month assessment, 1 had incomplete follow-up records, and 3 had insufficient preoperative or clinical documentation. In the LLS group, 3 patients were lost to follow-up, 2 had incomplete follow-up records, and 4 had insufficient preoperative or clinical documentation. The final analytical cohort therefore comprised 97 women, including 33 in the vNOTES-LS group and 64 in the LLS group (Figure 1).

All participants were postmenopausal. No statistically significant between-group differences were observed in the measured baseline demographic and clinical characteristics, including age, body mass index, parity, number of vaginal births, sexual activity, medical comorbidities, smoking status, previous prolapse or stress urinary incontinence surgery, preoperative POP-Q stage, or concomitant hysterectomy (Table 1). Individual baseline POP-Q measurements (Aa, Ba, C, D, Ap, Bp, gh, pb, and TVL) also showed no statistically significant between-group differences (all *p* > 0.05). These findings should not be interpreted as evidence of complete baseline comparability because treatment allocation was non-randomized.

The frequencies of concomitant anterior colporrhaphy, posterior colporrhaphy, transobturator tape placement, and perineoplasty were also not statistically significantly different between the groups.

### 3.2. Perioperative Outcomes

Mean operating time was significantly shorter in the vNOTES-LS group than in the LLS group (78.0 ± 16.6 vs. 110.0 ± 14.2 min; *p* < 0.001). Estimated blood loss was also lower following vNOTES-LS, with median values of 90 mL (interquartile range [IQR], 80–110 mL) and 115 mL (IQR, 97.5–132.5 mL), respectively (*p* = 0.003). Postoperative hemoglobin decrease was not statistically significantly different between the groups (median, 1.2 [IQR, 0.9–1.3] vs. 1.2 [IQR, 1.0–1.4] g/dL; *p* = 0.148).

Median hospital stay was 1 day (IQR, 1–2) following vNOTES-LS and 2 days (IQR, 2–3) following LLS (*p* < 0.001). The median 24-h visual analog scale pain score was also lower in the vNOTES-LS group (3 [IQR, 2–4] vs. 4 [IQR, 4–5]; *p* < 0.001).

After adjustment for age, body mass index, parity, preoperative POP-Q stage, concomitant hysterectomy, and additional pelvic floor procedures, the vNOTES-LS approach was associated with a 31.7-min shorter operating time compared with LLS (adjusted β, −31.7 min; 95% CI, −38.7 to −24.6; *p* < 0.001) (Table 2).

No intraoperative bladder, bowel, ureteric, or major vascular injury occurred in either group. No procedure required conversion to another surgical approach, and no patient required perioperative blood transfusion. No postoperative infection or unplanned readmission was recorded during the early postoperative period.

### 3.3. Anatomical Outcomes

Anatomical success at 6 months was achieved in all patients in both groups, and no apical, anterior, or posterior compartment recurrence was recorded at this assessment.

At 12 months, anatomical success was observed in 29 of 33 women (87.9%) following vNOTES-LS and 57 of 64 women (89.1%) following LLS (*p* = 1.000). The relative risk of anatomical success for vNOTES-LS compared with LLS was 0.99 (95% CI, 0.85–1.15). The absolute between-group risk difference was −1.2 percentage points (95% CI, −14.7 to 12.3 percentage points).

At 6 months, POP-Q stages 0 and I were observed in 16 (48.5%) and 17 (51.5%) patients in the vNOTES-LS group and in 31 (48.4%) and 33 (51.6%) patients in the LLS group, respectively (*p* = 0.996). At 12 months, POP-Q stages 0, I, and II were observed in 21 (63.6%), 7 (21.2%), and 5 (15.2%) patients following vNOTES-LS and in 39 (60.9%), 17 (26.6%), and 8 (12.5%) patients following LLS, respectively (*p* = 0.822). No stage III or IV prolapse was observed in either group at either follow-up assessment.

At 12 months, apical recurrence occurred in 1 patient (3.0%) following vNOTES-LS and 2 patients (3.1%) following LLS (*p* = 1.000). Anterior compartment recurrence occurred in 2 (6.1%) and 5 (7.8%) patients, respectively (*p* = 1.000), whereas posterior compartment recurrence occurred in 1 patient (3.0%) following vNOTES-LS and in no patient following LLS (*p* = 0.340). No statistically significant between-group difference was detected for any compartment-specific recurrence outcome.

### 3.4. Safety Outcomes

No patient required reoperation during the first 6 postoperative months. By the 6-month assessment, mesh exposure had been diagnosed in 3 of 33 women (9.1%) following vNOTES-LS and 2 of 64 women (3.1%) following LLS (*p* = 0.333). Pelvic pain was reported by 5 women (15.2%) in the vNOTES-LS group and 8 women (12.5%) in the LLS group (*p* = 0.758).

Among women who were sexually active at baseline, dyspareunia at 6 months was reported by 2 of 22 women (9.1%) following vNOTES-LS and 3 of 39 women (7.7%) following LLS (*p* = 1.000). De novo stress urinary incontinence occurred in 3 (9.1%) and 7 (10.9%) women, respectively (*p* = 1.000). Urgency was reported by 7 (21.2%) and 10 (15.6%) women (*p* = 0.576), whereas voiding dysfunction occurred in 3 (9.1%) and 5 (7.8%) women, respectively (*p* = 1.000).

By 12 months, reoperation for recurrent prolapse had been required in 2 women (6.1%) following vNOTES-LS and 1 woman (1.6%) following LLS (*p* = 0.266). The two reoperations in the vNOTES-LS group were associated with one apical and one anterior recurrence, whereas the reoperation in the LLS group was associated with apical recurrence. All three women subsequently underwent laparoscopic sacrocolpopexy.

Mesh exposure was diagnosed at some time during the first 12 postoperative months in 3 of 33 women (9.1%) following vNOTES-LS and 6 of 64 women (9.4%) following LLS (*p* = 1.000). Five exposures had been identified by the 6-month assessment. Four of these resolved by 12 months, whereas one exposure in the vNOTES-LS group persisted. Four additional exposures were newly diagnosed between 6 and 12 months, all in the LLS group. Consequently, active mesh exposure at the 12-month examination was present in 1 of 33 women (3.0%) following vNOTES-LS and 4 of 64 women (6.3%) following LLS (*p* = 0.659). Mesh exposures were diagnosed between postoperative months 3 and 12 and ranged from 0.5 to 1.5 cm in diameter. Seven exposures involved the vaginal cuff and two involved the anterior vaginal wall. Four women presented with dyspareunia, three with vaginal bleeding, and two were asymptomatic at the time the exposure was identified. Initial management consisted of vaginal estrogen in five women and mesh excision in four. All nine women who developed mesh exposure had undergone concomitant vaginal hysterectomy. The timing, size, location, management, and subsequent clinical course of the individual mesh exposure cases are summarized in Table 3.

At 12 months, pelvic pain was reported by 3 women (9.1%) following vNOTES-LS and 6 women (9.4%) following LLS (*p* = 1.000). Dyspareunia was reported by 2 of 22 sexually active women (9.1%) and 3 of 39 sexually active women (7.7%), respectively (*p* = 1.000). De novo stress urinary incontinence occurred in 2 (6.1%) and 5 (7.8%) women (*p* = 1.000), urgency in 6 (18.2%) and 7 (10.9%) women (*p* = 0.356), and voiding dysfunction in 1 (3.0%) and 3 (4.7%) women (*p* = 1.000), respectively. No statistically significant between-group differences were detected for these safety outcomes; however, the number of individual adverse events was small.

### 3.5. Patient-Reported Outcomes

PFDI-20 and PFIQ-7 scores improved from baseline at both 6 and 12 months in each group. A significant effect of time was observed for both PFDI-20 and PFIQ-7 (*p* < 0.001 for both). No statistically significant time-by-group interaction was detected for PFDI-20 (*p* = 0.992) or PFIQ-7 (*p* = 0.688), indicating that the available data did not demonstrate a differential trajectory of change between the two surgical approaches.

The mean 12-month change in PFDI-20 score was −62.7 ± 15.3 in the vNOTES-LS group and −62.6 ± 15.4 in the LLS group (*p* = 0.956). The corresponding changes in PFIQ-7 score were −69.6 ± 14.2 and −68.0 ± 13.2, respectively (*p* = 0.590).

PISQ-12 was evaluated in 61 women who were sexually active at baseline, including 22 in the vNOTES-LS group and 39 in the LLS group. PISQ-12 scores improved significantly over time in both groups (*p* < 0.001). No statistically significant time-by-group interaction was detected for PISQ-12 scores (*p* = 0.988), indicating that the available data did not demonstrate a differential trajectory of sexual-function improvement between the two surgical approaches.

At 6 months, a PGI-I score of 1 or 2 was reported by 23 of 33 women (69.7%) in the vNOTES-LS group and 47 of 64 women (73.4%) in the LLS group (*p* = 0.812). At 12 months, the corresponding proportions were 19 of 33 (57.6%) and 39 of 64 (60.9%), respectively (*p* = 0.828). Longitudinal changes in PFDI-20, PFIQ-7, and PISQ-12 scores are summarized in Table 4.

## 4. Discussion

In this retrospective cohort of women with advanced pelvic organ prolapse, observed 12-month anatomical and functional outcome measures were not statistically significantly different between vNOTES-LS and conventional LLS, whereas vNOTES-LS was associated with shorter operating time and favorable early perioperative outcomes. These findings should not be interpreted as demonstrating equivalence or non-inferiority between the procedures. In particular, the relatively wide confidence interval around the between-group difference in anatomical success indicates that clinically meaningful differences in either direction cannot be excluded. Anatomical success at 12 months exceeded 87% in both groups, with no significant differences in overall success, POP-Q stage distribution, compartment-specific recurrence, safety outcomes, or patient-reported improvement. In contrast, vNOTES-LS was associated with shorter operating time, lower estimated blood loss, shorter hospitalization, and lower pain scores at 24 h. The adjusted analysis further showed that shorter operating time remained associated with the vNOTES approach after adjustment for potential confounders.

The anatomical success rates observed in both groups are consistent with the contemporary evidence supporting lateral suspension for anterior and apical prolapse. A 2025 systematic review and meta-analysis including 18 studies and 1430 women reported pooled anatomical success rates of 92.9% for the apical compartment and 86.9% for the anterior compartment, together with an overall recurrence rate of 9.6% [12]. Similarly, Wang et al. reported 2-year objective cure rates of 100% for the apical compartment, 88.3% for the anterior compartment, and 93.3% for the posterior compartment following conventional LLS in women with severe prolapse [13]. Although our definition of anatomical success was based on the absence of prolapse reaching the hymen and the absence of reoperation, the 12-month success rates of 87.9% following vNOTES-LS and 89.1% following LLS fall within the range reported in these contemporary series. However, the observed proximity of these point estimates should not be interpreted as evidence of equivalent efficacy. The absolute risk difference was −1.2 percentage points, with a 95% confidence interval from −14.7 to 12.3 percentage points, reflecting substantial statistical uncertainty.

The most prominent difference between the surgical approaches was observed in perioperative recovery. The approximately 32-min difference in operating time remained evident after adjustment for age, body mass index, parity, preoperative POP-Q stage, concomitant hysterectomy, and additional pelvic floor procedures (adjusted β, −31.7 min; 95% CI, −38.7 to −24.6). Nevertheless, residual confounding cannot be excluded. The avoidance of conventional abdominal trocar placement, reduced abdominal wall manipulation, and direct transvaginal access to the pelvis may collectively account for the shorter operation, lower early pain scores, and shorter hospitalization. The shorter hospital stay observed in the present cohort is consistent with both comparative studies [5,6], whereas shorter operative time was reported in the prospective study by Sengi et al. [6] but not in the retrospective study by Uluutku Bulutlar et al. [5]. More recently, a prospective comparison of vNOTES-LS and laparoscopic sacrohysteropexy reported shorter operating time, lower postoperative pain scores, and shorter hospital stay with vNOTES-LS, while early apical success rates were 94% and 96% in the vNOTES-LS and laparoscopic sacrohysteropexy groups, respectively [14]. A 2026 retrospective comparison of single-port lateral suspension and multiport sacrohysteropexy likewise reported shorter apical operative time and hospital stay with single-port lateral suspension, while both groups showed improvement in short-term anatomical and functional outcomes [15]. Collectively, these findings support perioperative efficiency as the clearest potential advantage of the vNOTES route.

Surgeon experience and potential learning-curve effects should also be considered when interpreting the observed difference in operating time. All procedures in the present study were performed by the same surgeon, who had 11 years of specialist experience in obstetrics and gynecology and approximately 8 years of experience with conventional laparoscopic lateral suspension. In contrast, vNOTES-LS was introduced into his clinical practice in August 2024 and therefore represented a more recently adopted technique. Although the surgeon subsequently accumulated experience of approximately 70 vNOTES-LS procedures, temporal changes in procedural familiarity, operative standardization, case selection, and perioperative workflow may have influenced operative duration during the study period. Consequently, the approximately 32-min shorter operating time observed with vNOTES-LS should not be attributed exclusively to the route of surgical access, and this finding should be interpreted in the context of the single-surgeon design and potential temporal and learning-curve effects.

These potential perioperative advantages should, however, be considered alongside several procedure-specific limitations. vNOTES-LS requires familiarity with transvaginal endoscopic anatomy and a dedicated learning curve, and its feasibility may be influenced by pelvic anatomy, previous surgery, adhesions, and the ability to obtain safe transvaginal access. Although avoidance of conventional abdominal trocar placement may reduce abdominal wall trauma and early postoperative discomfort, vNOTES-LS remains a mesh-based reconstructive procedure and therefore retains mesh-related risks, including exposure, pain, and the potential need for subsequent intervention. In addition, differences in the route of mesh introduction, fixation, tissue coverage, and concomitant vaginal surgery may influence prosthesis-related outcomes. The present study was not designed to determine whether either route provides superior long-term mesh safety. Accordingly, selection of vNOTES-LS should currently be individualized according to patient anatomy, previous surgery, surgeon expertise, and patient preference rather than based solely on its observed perioperative profile.

Compartment-specific analysis provides additional clinical context. In the present cohort, anterior recurrence was the most frequent anatomical failure at 12 months, occurring in 6.1% of patients following vNOTES-LS and 7.8% following LLS. Apical recurrence remained uncommon, and posterior recurrence was identified in only one patient. This pattern is compatible with previous observations that lateral suspension provides particularly strong apical support, whereas the anterior compartment may remain the most vulnerable site during follow-up. In a continuous series of 417 patients, Veit-Rubin et al. reported 1-year anatomical success rates of 93.6% for the apex, 91.6% for the anterior compartment, and 85.3% for the posterior compartment [16]. Modifications incorporating additional posterior mesh arms have been proposed to improve multicompartment support, although these techniques introduce greater procedural and prosthetic complexity [17]. Posterior compartment failure was infrequent in this cohort; however, the limited number of events precludes conclusions regarding the adequacy of selective concomitant posterior repair.

No statistically significant between-group differences were detected in the assessed safety outcomes; however, the limited number of events restricts the precision of these comparisons, and the mesh exposure findings deserve particular consideration. Mesh exposure was diagnosed at some time during the first 12 postoperative months in 3 of 33 women (9.1%) following vNOTES-LS and 6 of 64 women (9.4%) following LLS. Importantly, these figures represent cumulative exposure detected during the first postoperative year rather than the prevalence of active exposure at the 12-month examination. At 12 months, active mesh exposure was present in 1 of 33 women (3.0%) following vNOTES-LS and 4 of 64 women (6.3%) following LLS. The cumulative mesh exposure rate observed in our cohort was substantially higher than the pooled overall mesh-related complication rate of 1.9% reported in the recent meta-analysis of LLS [12] and the 4.3% mesh exposure rate reported in the long-term series of Veit-Rubin et al. [16]. Although the 1.9% pooled estimate represents a broader category of mesh-related complications rather than mesh exposure alone, the marked numerical difference warrants attention. Differences in mesh characteristics, concomitant hysterectomy rates, exposure definitions, intensity of postoperative examination, and duration of surveillance may partly explain this variation. Notably, almost 90% of the women in both groups underwent concomitant hysterectomy. The high frequency of concomitant hysterectomy may have contributed to the observed exposure rate; however, the available comparative evidence relates primarily to supracervical hysterectomy and cannot be directly extrapolated to vaginal hysterectomy [18]. Accordingly, concomitant hysterectomy and vaginal cuff healing may represent potential contributing factors to the observed exposure rate, although the retrospective design does not permit causal attribution. Although the cumulative exposure rates observed within 12 months were numerically close between the groups, the small number of events precludes firm conclusions regarding comparative mesh-related safety. Nevertheless, longer surveillance and detailed reporting of exposure size, location, symptoms, management, persistence, and resolution will be important in future studies.

Reoperation was required in two women following vNOTES-LS and one woman following LLS. Although the difference was not statistically significant, the low number of events substantially limits statistical precision. Therefore, the absence of a significant difference should not be interpreted as evidence that reoperation risks are equivalent. The pooled reoperation rate after LLS has been estimated at approximately 6.2%, but considerable variation exists according to follow-up duration, recurrence definition, index prolapse pattern, and concomitant procedures [12]. The wide confidence interval surrounding the absolute difference in anatomical success in our study similarly indicates that clinically meaningful differences in either direction cannot be excluded. These results should therefore be interpreted only as an absence of a statistically detectable between-group difference in this limited cohort and not as evidence of equivalence or non-inferiority of vNOTES-LS.

Both procedures produced substantial and sustained improvements in pelvic floor symptom burden and quality of life. PFDI-20 and PFIQ-7 scores improved markedly at 6 months, with most of the benefit maintained at 12 months. No statistically significant time-by-group interaction was detected for these outcomes, indicating that the available data did not demonstrate a differential trajectory of improvement between the two approaches. These findings are consistent with the significant improvements in PFDI-20 and PFIQ-7 reported at 2 years after conventional LLS [13]. Recent comparative data have also demonstrated meaningful improvements in pelvic floor distress and sexual function after lateral suspension, with anatomical correction accompanied by improvement in patient-reported outcomes [19]. Functional outcomes improved over time in both groups, and the available data did not demonstrate a statistically significant differential trajectory between the two approaches; however, the study was not powered to exclude clinically meaningful between-group differences in these outcomes. These findings can also be considered within the broader literature on minimally invasive apical suspension. Pitsillidi et al. reported significant improvements in validated patient-reported quality-of-life and functional outcomes following laparoscopic pectopexy, further supporting the value of assessing symptom burden and patient-centered outcomes in addition to anatomical correction after prolapse surgery [20]. Although pectopexy and lateral suspension differ in their fixation sites and operative techniques, these findings provide useful contextual evidence for the improvements observed in PFDI-20, PFIQ-7, and PISQ-12 in the present cohort.

Sexual function also improved significantly over time in both groups, with no evidence of a differential treatment effect. This analysis was appropriately restricted to women who were sexually active at baseline and had complete assessments at all three time points. Although this restriction reduces the available sample size, it avoids assigning PISQ-12 values to women for whom the questionnaire is not applicable. No statistically significant between-group difference in postoperative dyspareunia was detected; however, the relatively small sexually active subgroup and low number of events limit the precision of this comparison.

Anatomical success and patient-perceived global improvement were not fully concordant. Despite anatomical success rates approaching 90%, PGI-I subjective success at 12 months was reported by approximately 58% of the vNOTES-LS group and 61% of the LLS group. These proportions are lower than the pooled subjective success rate of 88.9% reported for LLS [12]; however, direct comparison should be interpreted cautiously because definitions and assessment methods for subjective success may differ across studies. Several factors may account for this difference. First, the present study used a relatively strict definition of subjective success, requiring a PGI-I score of 1 or 2. Second, patient-perceived improvement may be influenced by urinary urgency, stress urinary incontinence, pelvic pain, dyspareunia, mesh exposure, bowel symptoms, and preoperative expectations, even when anatomical support is satisfactory. The modest increase in PFDI-20 and PFIQ-7 scores between 6 and 12 months in both groups may likewise indicate partial symptom recurrence or adaptation of patient expectations without corresponding advanced anatomical recurrence. These findings emphasize the importance of reporting anatomical and patient-centered outcomes separately.

The current evidence supporting vNOTES-based apical suspension remains encouraging but relatively immature. A recent systematic review of vNOTES uterosacral ligament suspension found favorable short-term efficacy and safety but emphasized that most available studies were small, heterogeneous, and limited by short follow-up [21]. Similar limitations apply to the evolving literature on vNOTES-LS. The present study extends this evidence by providing a direct comparison with conventional LLS, complete 12-month anatomical assessment, compartment-specific recurrence data, validated patient-reported outcomes, and evaluation of mesh-related and urinary complications. However, definitive assessment of durability will require multicenter prospective studies with longer follow-up and adequately powered noninferiority designs.

Several strengths of this study should be acknowledged. Consecutive patients were treated at the same tertiary center by a single experienced surgeon, reducing variability related to surgical technique and perioperative management. Both anatomical and functional outcomes were assessed using standardized POP-Q measurements and validated questionnaires. No statistically significant between-group differences were observed in the measured major baseline and concomitant surgical characteristics, and the association between surgical approach and operating time was examined using multivariable adjustment. Reporting relative and absolute effect estimates with confidence intervals also provides greater clinical information than reliance on *p* values alone.

The study has several important limitations. First, its retrospective and non-randomized design introduces the possibility of selection bias and confounding by indication. Surgical route was determined according to pelvic anatomy, previous surgery, patient preference, and surgeon judgment rather than random allocation. Accordingly, the absence of statistically significant differences in measured baseline characteristics should not be interpreted as evidence that the groups were fully comparable or exchangeable. Unmeasured or incompletely measured factors, including prolapse morphology, pelvic accessibility, adhesions, and other elements influencing surgical decision-making, may have affected treatment selection and outcomes. Although multivariable adjustment was used for the analysis of operating time, residual confounding cannot be excluded. Because only 11 anatomical failures occurred, adequate multivariable adjustment of the primary anatomical outcome was not feasible without a substantial risk of overfitting and unstable effect estimates. Second, the single-center, single-surgeon setting provides technical consistency but limits the generalizability of the findings to other institutions and to surgeons at different stages of the vNOTES learning curve. Because postoperative POP-Q assessments were performed by the operating surgeon rather than by an independent examiner, observer-related assessment bias cannot be excluded. Because the final analysis excluded patients who were lost to follow-up or had insufficient preoperative or follow-up documentation, attrition-related and complete-case selection bias cannot be excluded. The characteristics and outcomes of excluded patients could not be fully compared with those of the analyzed cohort because complete outcome data were unavailable. Furthermore, the study was not designed or powered as a non-inferiority or equivalence trial, and the relatively small number of recurrence, reoperation, and complication events resulted in limited statistical precision. The high frequency of concomitant hysterectomy limits extrapolation to uterus-preserving lateral suspension. Finally, the 12-month follow-up period permits assessment of short-term anatomical and functional outcomes but is insufficient to establish long-term durability or to fully characterize late prolapse recurrence, mesh contraction, delayed mesh exposure, or the subsequent need for reoperation.

## 5. Conclusions

vNOTES-LS was associated with shorter operating time, lower estimated blood loss, reduced early postoperative pain, and shorter hospitalization compared with conventional LLS. Observed 12-month anatomical success, recurrence, safety, and patient-reported outcome rates were not statistically significantly different between the groups; however, the limited sample size and wide confidence intervals preclude conclusions regarding equivalence or non-inferiority. The mesh exposure rate observed in both groups also warrants continued surveillance. Larger multicenter prospective studies with adequate statistical power, standardized outcome definitions, detailed mesh-related reporting, and longer follow-up are required to establish comparative durability and safety.

## Figures and Tables

**Table 1:** Baseline demographic, clinical, and surgical characteristics.

Characteristic	vNOTES-LS (*n* = 33)	LLS (*n* = 64)	*p* Value
Age, years	62.61 ± 7.81	63.05 ± 7.46	0.790
Body mass index, kg/m^2^	27.6 (25.8–30.5)	27.3 (25.8–29.9)	0.790
Parity	3 (2–4)	3 (2–4)	0.988
Vaginal births	3 (2–4)	3 (2–4)	0.386
Sexually active	22 (66.7)	39 (60.9)	0.660
Diabetes mellitus	2 (6.1)	5 (7.8)	1.000
Hypertension	15 (45.5)	28 (43.8)	1.000
Constipation	9 (27.3)	18 (28.1)	1.000
Current smoking	6 (18.2)	7 (10.9)	0.356
Previous prolapse surgery	2 (6.1)	5 (7.8)	1.000
Previous SUI surgery	6 (18.2)	9 (14.1)	0.768
POP-Q stage IV	14 (42.4)	23 (35.9)	0.660
Baseline POP-Q measurements			
Aa, cm	2.06 ± 0.50	2.08 ± 0.45	0.865
Ba, cm	5.30 ± 1.10	5.36 ± 1.13	0.814
C, cm	3.27 ± 1.89	3.05 ± 1.87	0.578
D, cm	0.39 ± 0.66	0.30 ± 0.66	0.494
Ap, cm	0.18 ± 0.39	0.23 ± 0.43	0.546
Bp, cm	1.18 ± 1.18	1.06 ± 1.15	0.637
gh, cm	5.91 ± 0.58	5.83 ± 0.63	0.529
pb, cm	2.73 ± 0.45	2.64 ± 0.48	0.386
TVL, cm	9.39 ± 0.61	9.36 ± 0.65	0.797
Concomitant hysterectomy	29 (87.9)	58 (90.6)	0.731
Anterior colporrhaphy	3 (9.1)	5 (7.8)	1.000
Posterior colporrhaphy	2 (6.1)	2 (3.1)	0.603
Transobturator tape	1 (3.0)	3 (4.7)	1.000
Perineoplasty	1 (3.0)	3 (4.7)	1.000

Values are presented as mean ± standard deviation, median (interquartile range), or *n* (%). LLS, laparoscopic lateral suspension; POP-Q, Pelvic Organ Prolapse Quantification; SUI, stress urinary incontinence; TVL, total vaginal length; vNOTES-LS, vaginal natural orifice transluminal endoscopic surgery lateral suspension. POP-Q measurements are expressed in centimeters relative to the hymen.

**Table 2:** Perioperative, anatomical, 12-month safety, and global improvement outcomes.

Outcome	vNOTES-LS (*n* = 33)	LLS (*n* = 64)	*p* Value
Operating time, min	78.0 ± 16.6	110.0 ± 14.2	<0.001
Estimated blood loss, mL	90 (80–110)	115 (97.5–132.5)	0.003
Hemoglobin decrease, g/dL	1.2 (0.9–1.3)	1.2 (1.0–1.4)	0.148
Hospital stay, days	1 (1–2)	2 (2–3)	<0.001
VAS pain score at 24 h	3 (2–4)	4 (4–5)	<0.001
Anatomical success at 6 months	33 (100)	64 (100)	1.000
Anatomical success at 12 months	29 (87.9)	57 (89.1)	1.000
Apical recurrence at 12 months	1 (3.0)	2 (3.1)	1.000
Anterior recurrence at 12 months	2 (6.1)	5 (7.8)	1.000
Posterior recurrence at 12 months	1 (3.0)	0 (0)	0.340
Reoperation for recurrent prolapse within 12 months	2 (6.1)	1 (1.6)	0.266
Mesh exposure diagnosed within 12 months	3 (9.1)	6 (9.4)	1.000
Active mesh exposure at the 12-month examination	1 (3)	4 (6.3)	0.659
Pelvic pain at 12 months	3 (9.1)	6 (9.4)	1.000
Dyspareunia at 12 months *	2/22 (9.1)	3/39 (7.7)	1.000
De novo SUI at 12 months	2 (6.1)	5 (7.8)	1.000
Urgency at 12 months	6 (18.2)	7 (10.9)	0.356
Voiding dysfunction at 12 months	1 (3.0)	3 (4.7)	1.000
PGI-I subjective success at 12 months	19 (57.6)	39 (60.9)	0.828

Values are presented as mean ± standard deviation, median (interquartile range), or *n* (%). * Dyspareunia was evaluated only among women who were sexually active at baseline. SUI, stress urinary incontinence; VAS, visual analog scale. No compartment-specific recurrence or reoperation was recorded in either group at 6 months. Mesh exposure diagnosed within 12 months represents cumulative exposure identified at any time during the first postoperative year, whereas active mesh exposure at the 12-month examination represents exposure still present at the 12-month assessment.

**Table 3:** Clinical characteristics and course of mesh exposure cases.

Case	Procedure	Diagnosis, Postoperative Month	Exposure Size, cm	Location	Associated Symptoms	Status at 12 Months	Management
1	vNOTES-LS	4	0.5	Vaginal cuff	Dyspareunia	Resolved by 12 mo	Vaginal estrogen
2	vNOTES-LS	4	1.0	Vaginal cuff	Vaginal bleeding	Resolved by 12 mo	Vaginal estrogen
3	vNOTES-LS	6	0.5	Anterior vaginal wall	Asymptomatic	Persistent at 12 mo	Vaginal estrogen
4	LLS	3	1.5	Vaginal cuff	Vaginal bleeding	Resolved by 12 mo	Mesh excision
5	LLS	4	1.0	Vaginal cuff	Dyspareunia	Resolved by 12 mo	Mesh excision
6	LLS	8	1.0	Vaginal cuff	Vaginal bleeding	Active at 12 mo; newly diagnosed between 6–12 mo	Mesh excision
7	LLS	12	0.5	Vaginal cuff	Dyspareunia	Active at 12 mo; newly diagnosed between 6–12 mo	Vaginal estrogen
8	LLS	12	0.5	Vaginal cuff	Asymptomatic	Active at 12 mo; newly diagnosed between 6–12 mo	Vaginal estrogen
9	LLS	10	1.0	Anterior vaginal wall	Dyspareunia	Active at 12 mo; newly diagnosed between 6–12 mo	Mesh excision

LLS, laparoscopic lateral suspension; vNOTES-LS, vaginal natural orifice transluminal endoscopic surgery lateral suspension.

**Table 4:** Longitudinal patient-reported outcomes.

Outcome	vNOTES-LS Baseline/6 Months/12 Months	LLS Baseline/6 Months/12 Months	Time *p* Value	Time × Group *p* Value
PFDI-20	125.05 ± 14.32 55.35 ± 19.16 62.31 ± 20.86	124.00 ± 15.67 54.62 ± 21.50 61.44 ± 22.78	<0.001	0.992
PFIQ-7	110.27 ± 27.19 37.96 ± 24.95 40.67 ± 25.79	106.20 ± 28.24 35.97 ± 27.53 38.21 ± 28.04	<0.001	0.688
PISQ-12 *	23.96 ± 4.19 31.28 ± 4.92 31.04 ± 5.64	24.31 ± 4.44 31.74 ± 4.98 31.45 ± 5.70	<0.001	0.988

Values are presented as mean ± standard deviation. * PISQ-12 was analyzed among women who were sexually active at baseline and had complete measurements at all three time points (vNOTES-LS, *n* = 22; LLS, *n* = 39). Lower PFDI-20 and PFIQ-7 scores indicate lower symptom burden and less impairment, whereas higher PISQ-12 scores indicate better sexual function.

## Data Availability

The data supporting the findings of this study are available from the corresponding author upon reasonable request. The data are not publicly available owing to privacy and ethical restrictions.
